# Immunotherapy and the Combination with Targeted Therapies for Advanced Hepatocellular Carcinoma

**DOI:** 10.3390/cancers15030654

**Published:** 2023-01-20

**Authors:** Carmelo Laface, Girolamo Ranieri, Felicia Maria Maselli, Francesca Ambrogio, Caterina Foti, Michele Ammendola, Marigia Laterza, Gerardo Cazzato, Riccardo Memeo, Giovanni Mastrandrea, Marco Lioce, Palma Fedele

**Affiliations:** 1 Medical Oncology, Dario Camberlingo Hospital, 72021 Francavilla Fontana, Italy; 2 IRCCS Istituto Tumori “Giovanni Paolo II”, 70124 Bari, Italy; 3 Section of Dermatology, Department of Biomedical Science and Human Oncology, University of Bari, 70124 Bari, Italy; 4 Department of Health Science, General Surgery, Medicine School of Germaneto, Magna Graecia University, 88100 Catanzaro, Italy; 5 Division of Cardiac Surgery, University of Bari, 70124 Bari, Italy; 6 Department of Emergency and Organ Transplantation, Pathology Section, University of Bari “Aldo Moro”, Piazza Giulio Cesare 11, 70124 Bari, Italy; 7 Unit of Hepato-Pancreatic-Biliary Surgery, “F. Miulli” General Regional Hospital, 70021 Acquaviva Delle Fonti, Italy

**Keywords:** immunotherapy, targeted therapy, tumor microenvironment, hepatocellular carcinoma

## Abstract

**Simple Summary:**

One of the most important abilities of a tumor is to establish a state of immunosuppression inside the tumor microenvironment. This is made possible through numerous mechanisms of tumor immune escape that have been identified in experimental studies during the last decades. With regards to the liver, the hepatic microenvironment is commonly oriented towards a state of immune tolerance, preventing an autoimmune reaction. Moreover, since the etiology of Hepatocellular Carcinoma (HCC) is often related to cirrhosis, hepatitis B, or C, this tumor develops in the context of chronic inflammation. Given these data and the poor prognosis of advanced HCC, different immunotherapeutic strategies have been developed and evaluated for these patients. In this review, we describe all the clinical applications of immunotherapy for advanced HCC, from the drugs that have already been approved to the ongoing clinical trials.

**Abstract:**

One of the most important abilities of a tumor is to establish a state of immunosuppression inside the tumor microenvironment. This is made possible through numerous mechanisms of tumor immune escape that have been identified in experimental studies during the last decades. In addition, the hepatic microenvironment is commonly oriented towards a state of immune tolerance because the liver receives blood from the hepatic arteries and portal veins containing a variety of endogenous antigens. Therefore, the hepatic microenvironment establishes an autoimmune tolerance, preventing an autoimmune reaction in the liver. On this basis, hepatic tumor cells may escape the immune system, avoiding being recognized and destroyed by immune cells. Moreover, since the etiology of Hepatocellular Carcinoma (HCC) is often related to cirrhosis, and hepatitis B or C, this tumor develops in the context of chronic inflammation. Thus, the HCC microenvironment is characterized by important immune cell infiltration. Given these data and the poor prognosis of advanced HCC, different immunotherapeutic strategies have been developed and evaluated for these patients. In this review, we describe all the clinical applications of immunotherapy for advanced HCC, from the drugs that have already been approved to the ongoing clinical trials.

## 1. Introduction

In the last years, the deepening of knowledge about the mechanisms of tumor immune escape led to the development of immunological drugs for the treatment of various cancers with favorable results [1,2,3,4]. In general, tumor immunotherapy is based on the exaltation of the immune response to enhance the immune cells’ antitumor activity and overcome the cancer immune escape [5].

One of the most important abilities of a tumor is to establish a state of immunosuppression inside the tumor microenvironment (TME). This is made possible through numerous mechanisms of immune escape that have been identified in experimental studies during the last decades [1,2,3,4]. In this regard, regulatory T cells (Tregs) and myeloid-derived suppressor cells (MDSCs) are the most important immunosuppressive cells [2,3,4,6]. In addition, M2-polarized tumor-associated macrophages can also favor a state of immunosuppression in TME.

Immune checkpoints (ICPs) are immunosuppressive molecules including cytotoxic T lymphocyte-associated antigen 4 (CTLA-4), programmed cell death 1 (PD-1) receptor and its ligands PD-L1 and PD-L2, lymphocyte activation gene-3 (LAG-3), and T-cell immunoglobulin mucin-3 (TIM-3) [7,8,9]. ICPs are expressed on the surface of several immune cells including dendritic cells (DC), B and T cells, natural killer cells (NKs), monocytes, and tumor-associated macrophages (TAMs) [7,8,9]. ICPs physiologically inhibit the activation of these immune cells, avoiding an autoimmune reaction. CTLA-4 is expressed on DCs, Tregs, and activated T cells. It is a ligand for B7 (CD80/CD86), a transmembrane protein receptor expressed on the antigen-presenting cell (APC) membrane [10,11,12]. CTLA-4 competes with CD28 for B7 binding counteracting T cell co-stimulation. Therefore, the complex of CTLA-4 with B7 prevents the binding of CD28 to B7 with the consequent inhibition of T cells [12]. CTLA-4 can also enhance Tregs activity and differentiation [10]. PD-1 is a T-cell, B-cell, NK, DC, and MDSC membrane receptor [13]. PD-L1 and PD-L2 are the ligands of PD-1 and are expressed on various cells: APC, macrophages, parenchymal and cancer cells, and hematopoietic stem cells [14]. These molecules belong to the family of B7 transmembrane protein receptors, and their binding to PD-1 causes the inhibition of immune cell proliferation and activity [13,15].

The hepatic microenvironment is commonly oriented towards a state of immune tolerance because the liver receives blood from the hepatic arteries and portal veins containing a variety of endogenous antigens and autoantigens, respectively [16]. Therefore, the hepatic microenvironment establishes an autoimmune tolerance, preventing an autoimmune reaction in the liver. On this basis, hepatic tumor cells may escape the immune system, avoiding being recognized and destroyed by immune cells. Moreover, since the etiology of Hepatocellular Carcinoma (HCC) is often related to cirrhosis and hepatitis B or C, this tumor develops in the context of chronic inflammation [17,18,19,20]. Thus, the HCC microenvironment is characterized by important immune cell infiltration. In detail, high levels of immune inhibitory cells in the TME such as tumor-infiltrating lymphocytes (TILs) and Tregs may favor the expression of transforming growth factor-β (TGF-β) or increase the levels of CTLA4 and PD-L1, suppressing the immune response in patients affected by HCC [21,22]. Although M1 macrophages are known for exerting an anti-cancer role, they too may favor PD-L1 expression in hepatic tumor cells [23]. The same tumor cells can express a high level of PD-1 ligands [24]. MDSCs can release immunosuppressive cytokines, IL-10, and TGF-β, with the subsequent suppression of T-cell activation and upregulation of Tregs [25,26]. Furthermore, clinical studies have demonstrated that a poorer prognosis, tumor progression, and invasion are related to high levels of ICPs because of their ability to generate immune escape. On the other hand, experimental data from HCC models have put evidence that the suppression of ICPs can inhibit HCC growth [27,28]. It was well-defined that tumor-associated antigens (TAAs) must be processed before being presented to cytotoxic T-lymphocytes (CTLs) through major histocompatibility complex class 1 (MHC-1). The antigen recognition led to the activation of the immune response by CTLs. However, clinical data have documented that some cytokines, such as IL-1, −4, and −5, are overexpressed in HCC leading to a higher ratio of CD4+ to CD8+ T cells and a lower expression of MHC-1 [29]. This condition can prevent TAAs recognition by CTLs and favor immune escape. The treatment strategy for HCC depends on its stage. Several staging systems are currently employed in clinical practice, although the Barcelona Clinic HCC (BCLC) system is the most commonly used [30]. It is based on performance status (PS), tumor size and number, and liver function by means of Child–Pugh score [30,31]. Surgical resection, liver transplantation, and loco-regional therapies (radiofrequency, transarterial radioembolization, or chemoembolization) are the standard treatments for early stage [32,33,34,35,36,37]. However, 70% of early HCC patients experience a disease recurrence in the following 5 years. Moreover, only 30% of cases are diagnosed in the early stages, because of the silent clinical history.

On the basis of the reported data, given the poor prognosis of advanced HCC, different immunotherapeutic strategies have been developed and evaluated in clinical trials for these patients. Nowadays, new treatment chances are available. Therefore, in this review, we describe all the clinical applications of immunotherapy for advanced HCC, from the drugs that have been already approved to the ongoing clinical trials.

## 2. PD-1/PD-L1 Inhibitors

Chronic inflammation of the liver, related to viral and non-viral etiologies, is the main risk factor for HCC development [38]. Pro-inflammatory cytokines lead to high expression of PD-1 on TILs and of PD-L1 and PD-L2 on Kupffer cells, sinusoidal endothelial cells, and leukocytes [39].

Immune checkpoint inhibitors (ICIs) avoid the binding of PD-L1 and PD-L2 to PD-1 preventing the immune escape and promoting the recognition and killing of cancer cells by the immune response [39].

Therefore, the clinical evaluation of PD-1 and PD-L1 inhibitors for advanced HCC patients took place. Table 1 summarizes PD-1/PD-L1 inhibitors that have been tested for the treatment of advanced HCC.

### 2.1. Single Agents

#### 2.1.1. Nivolumab

Nivolumab (Opdivo) is the first fully humanized IgG4 monoclonal antibody (mAb) targeting PD-1. In this way, it blocks T-cell inhibition mediated by the PD-1/PD-L1 and PD-L2 pathway restoring anticancer immune activity [48].

Checkmate 040 was the first phase I/II trial that, for the first time, evaluated the safety and efficacy of Nivolumab on 262 aHCC patients, irrespective of prior therapies (Sorafenib or others) and HBV/HCV infection [40]. A good liver function was required (Child–Pugh score of ≤7) as well as the ECOG performance status (ECOG PS ≤ 1). The results put in evidence an objective response rate (ORR) of 15–20%, a disease control rate (DCR) of 58–64%, a median time to progression (mTTP) of 3.4 months, and overall survival (OS) of 15 months. Interestingly, the aHCC patients that received Sorafenib as first-line treatment also had good outcomes: ORR of 19% and OS of 13.2 months. Further analysis of CheckMate 040 Cohort 5 showed that Nivolumab is also efficient for patients with Child–Pugh B showing an ORR of 12% and a DCR of 55%; safety was also acceptable [49]. Note that the sub-analyses of CheckMate-040 demonstrated that a subgroup of patients treated with nivolumab experienced disease progression although they obtained a nonconventional benefit. In terms of toxicity, grade 3/4 adverse events (AEs) were experienced by 25% of patients [50].

On this basis, in 2017 Nivolumab was approved by the FDA as second-line therapy for aHCC patients who progressed to Sorafenib.

Subsequently, Checkmate 459, a randomized multicenter phase III clinical trial compared Nivolumab (240 mg intravenously every 2 weeks) to Sorafenib (400 mg orally twice daily) as first-line treatment in 743 aHCC patients, irrespectively of viral hepatitis status. They were no more suitable for surgical resection or locoregional treatments and had not previously received systemic therapy for this tumor. Child–Pugh class A and ECOG PS score of 0–1 criteria were required. Respectively, OS was 16.4 months and 14.7 months for the Nivolumab group and control one (HR = 0.85; *p* = 0.0752), but without a statistically significant difference. ORR was higher for the experimental group (15% vs. 7%). As regards safety, patients in the Nivolumab group experienced a lower incidence of grade 3/4 AEs (22% vs. 49%) compared to Sorafenib one. The most frequent severe AEs were palmar-plantar erythrodysesthesia (<1% in the experimental group vs. 14% in the control group), aspartate aminotransferase elevation (6% vs. 4%), and hypertension (0 vs. 7%) [41].

Recently, some studies also compared Nivolumab to Regorafenib in aHCC patients after progression to Sorafenib. These trials demonstrated a better ORR and lower AEs in the Nivolumab group [51,52].

Therefore, Nivolumab might be useful as a first- or second-line therapy for these patients.

#### 2.1.2. Pembrolizumab

Pembrolizumab is another anti-PD-1 IgG4 mAb able to block T-cell inhibition mediated by the PD-1/PD-L1 and PD-L2 pathway restoring anticancer immune activity [48]. The KEYNOTE-224 was the first phase II trial that tested Pembrolizumab (200 mg intravenously every 3 weeks for about 2 years) as second-line therapy for 104 aHCC patients after Sorafenib treatment. An ECOG performance status of 0–1 and a Child–Pugh class A were required. The study reported an ORR of 17%, a median OS of 12.9 months, and progression-free survival (PFS) of 4.9 months. Grade 3–4 treatment-related AES occurred in 25% of patients: increased aspartate aminotransferase level (7%) or alanine aminotransferase (4%), and fatigue (4%) [42].

On this basis, a randomized, double-blind, placebo-controlled phase III trial, KEYNOTE-240, was designed to confirm the efficacy and safety of Pembrolizumab (200 mg intravenously every 3 weeks for 2 years) with respect to placebo in 413 aHCC patients after first-line with Sorafenib. An ECOG performance status of 0–1 and a Child–Pugh class A were required. A median OS of 13.9 months versus 10.6 months (HR, 0.781; *p* = 0.0238), and a PFS of 3.0 months versus 2.8 months (HR, 0.718; *p* = 0.0022) were reported for the experimental and control group, respectively. However, despite the encouraging results, the primary endpoints have not reached the predetermined statistical significance (*p* = 0.0174). An ORR of 18.3% and 4.4% were observed for the pembrolizumab group and placebo one (*p* = 0.00007), respectively. A post hoc analysis of this study demonstrated that Pembrolizumab did not significantly compromise hepatic function with respect to placebo and that the survival improvement was independent of ALBI grade. Grade 3–4 AEs were reported in 52.7% and 46.3% for the experimental and control group, respectively [43].

KEYNOTE-394 is a phase III study conducted in Asia on patients previously treated for aHCC comparing Pembrolizumab (200 mg intravenously every 3 weeks for 2 years) with placebo. An ECOG performance status of 0–1 and a Child–Pugh class A were required. The trial put in evidence a significant survival improvement (13.6 vs. 13.0 months; HR 0.79, *p* = 0.0180) as well as PFS (2.6 vs. 2.3 months; HR 0.74, *p* = 0.0032), reaching the prespecified statistical criteria. ORR was 13.7% vs. 1.3% and median TTP was 2.7 vs. 1.7 months (HR 0.72) for the experimental and control group, respectively. Patients in the experimental group experienced a higher incidence of grade 3/4 AEs (14.4% and 5.9%) [44]. Therefore, the results were in line with KEYNOTE-224 and KEYNOTE-240 data.

Currently, a clinical trial (NCT04442581) is ongoing to compare Cabozantinib with Pembrolizumab as a first-line treatment.

Considering the reported data, in 2018 the FDA approved Pembrolizumab as a second-line therapy option for aHCC patients who previously progressed or experienced severe toxicity with Sorafenib.

#### 2.1.3. Other PD-1/PD-L1 Inhibitors

Besides Nivolumab and Pembrolizumab, some other anti-PD-1 mAb were tested in clinical trials to evaluate their antitumor activity in aHCC.

A multicenter, open-label, randomized, phase II trial analyzed the safety and the anticancer activity of Camrelizumab (3 mg/kg intravenously every 2 or 3 weeks), a PD-1 inhibitor. The study enrolled 220 Chinese patients affected by aHCC who received or were intolerant to previous systemic therapy. An ECOG performance status of 0–1 and a Child–Pugh class A were required. An ORR of 14.7% and an OS of 74.4% at six months were observed. Grade 3/4 AEs, like increased AST and decreased neutrophils, were experienced in 22% of patients [45].

Tislelizumab, a PD-1 inhibitor, documented an antitumor activity for pretreated aHCC patients in phase I and II trials, with a safety profile. On this basis, the randomized phase III RATIONALE-301 trial tested Tislelizumab (200 mg IV Q3W) against Sorafenib (400 mg PO BID) as a first-line treatment in 674 aHCC patients. The results demonstrated the OS non-inferiority of Tislelizumab compared to Sorafenib (15.9 vs. 14.1 months; HR: 0.85). In addition, Tislelizumab led to higher ORR (14.3% vs. 5.4%) and more durable responses (36.1 vs. 11.0 months) with respect to the control group. Grade ≥ 3 AEs occurred in 48.2% and 65.4% for Tislelizumab and Sorafenib, respectively [46].

Finally, Durvalumab was tested on aHCC patients previously treated with Sorafenib in a phase I/II clinical study. Of note, the major benefit of Durvalumab treatment regarded those patients affected by HCV infection in terms of survival (OS 19.3 vs. 13.2 months) and ORR (25% vs. 10.3%) [47].

### 2.2. PD-1/PD-L1 Inhibitors plus Antiangiogenetic Therapies

Several studies described the immunosuppressive role of pro-angiogenetic factors in three different ways. First, Treg cell proliferation and their homing to TME can be stimulated by VEGF [53,54]. This latter is also able to inhibit DC maturation, CD8+ T cell proliferation and action, and to promote T cell exhaustion by upregulating PD-1 expression on T cells [32,34,37,55,56,57,58,59,60].

On the other hand, Angiopoietin 2 (ANG2) can favor immunosuppression by binding to macrophages and monocytes while HGF and PDGFAB can suppress DC maturation. In addition, HGF can also suppress T-cell function. Second, the expression of some adhesion molecules on endothelial cells can allow the TME infiltration by certain immunosuppressive cells (for example, stabilin 1-mediated T_reg_ cell trafficking) or block TME infiltration by certain effector cells (for example, intercellular adhesion molecule 1 (ICAM1) downregulation leads to the suppression of NK cell and T cell trafficking). Third, vascular normalization can result in increased immune-cell infiltration and reduced hypoxia. The VEGF or ANG2 blockade can result in the transient normalization of the aberrant tumor vasculature with more-regular vessel patterning and pericyte coverage. On this basis, anti-angiogenetic therapy can lead to antitumor activity also through the modulation of the immune system into TME. However, the only blockade of angiogenic factors is insufficient to generate an important immune response against tumor, hence the need to combine anti-angiogenetic drugs with immunotherapies to boost adaptive immune responses [32,34,37,55,56,57,58,59,60]. As well as emerging data suggest the potential immunomodulatory role in TME of anti-angiogenic agents, on the other hand several studies showed that immunotherapeutic drugs might also improve the efficacy of anti-angiogenic agents or have more potent effects on changes to the tumor vasculature. In detail, anti-angiogenics can lead to increased effector immune-cell infiltration (such as CD8+ T cells or NK cells) in TME by inducing vessel normalization and/or relieving immunosuppression while immunotherapies can further activate effector immune cells or reinvigorate effector cells that have been suppressed (for example, by immune-checkpoint molecules). Activated immune effector cells in TME can secrete IFNγ, which can promote vascular remodeling [61].

The combination of anti-angiogenetics with immunotherapies has been already evaluated in several cancers including advanced HCC with interesting results.

Table 2 summarizes all the combinations of PD-1/PD-L1 inhibitors with angiogenetic drugs that have been tested for the treatment of advanced HCC.

#### 2.2.1. Atezolizumab and Bevacizumab

IMBrave150 [62] is a phase III study that analyzed Atezolizumab (anti-PD-L1 mAb) in combination with Bevacizumab (anti-VEGF mAb) with respect to Sorafenib as first-line treatment for naïve patients who suffered from aHCC [62]. The experimental group obtained a significant survival improvement in comparison with the control group (19.2 vs. 13.4 months; HR = 0.66; *p* = 0.0009) as well as PFS (6.8 months vs. 4.3 months; HR 0.59) and ORR (29.8% vs. 11.3%). No significant difference was reported between the two groups in terms of toxicity (56.5% vs. 55.1%). In this regard, hypertension and increased AST or ALT were the most frequent grade 3/4 AEs. On the basis of these favorable data, the experimental combination was approved as first-line therapy for this set of patients.

#### 2.2.2. Sintilimab and Bevacizumab

ORIENT-32 is a randomized, open-label, phase II/III clinical trial designed to analyze Sintilimab (Tyvyt) (a PD-1 inhibitor) (200 mg every 3 weeks) plus IBI305 (a bevacizumab biosimilar) (15 mg/kg every 3 weeks) in comparison with Sorafenib (400 mg orally twice daily) for 595 patients suffering from HBV-associated aHCC who did not receive prior systemic treatment. Patients in the experimental group experienced a significantly longer median PFS (4.6 months vs. 2.8 months, HR 0.56, *p* < 0.0001) as well as OS at the first interim analysis (median not reached vs. 10.4 months; HR 0.57; *p* < 0.0001). Among grade 3/4 AEs, hypertension was more frequent in the Sintilimab–bevacizumab biosimilar group (14% versus 6%) unlike palmar-plantar erythrodysesthesia syndrome (0% versus 12%) [63].

Therefore, this combination proved to be safer and more efficient than Sorafenib as first-line treatment for Chinese patients with HBV-associated aHCC and might provide a novel therapeutic option for these patients.

#### 2.2.3. Pembrolizumab and Lenvatinib

Lenvatinib (Lenviva) is a selective, multi-targeted TKI of VEGFR 1–3, FGFR 1–4, PDGFRα, RET, and KIT. It has been demonstrated that this drug can inhibit the immunosuppressive effects of TME. This action can be used to enhance the PD-1 antibodies activity, by increasing the number of CD8+ T cells as described in an HCC model. In this regard, the combination of Lenvatinib with an anti-PD1, such as Pembrolizumab, has been already evaluated in advanced endometrial cancer patients with favorable results.

On this basis, this combination was also tested in a phase Ib study for 104 patients with aHCC. The trial reported a median OS, PFS, and ORR of 22 months, 9.3 months, and 46%, respectively. Grade 3/4 AEs were observed in 67% of patients [68].

Considering these encouraging data, the double-blind randomized controlled phase III LEAP002 study was designed to compare this combination (Lenvatinib at 8 mg/day if BW < 60 kg or 12 mg/day if BW ≥ 60 kg plus Pembrolizumab at 200 mg IV Q3W) with Lenvatinib as first-line treatment for 794 patients. Results showed a median OS of 21.2 and 19 months for the experimental group and control group, respectively (HR 0.840, *p* = 0.0227). HR for PFS at interim analyses was 0.867 (*p* = 0.0466). ORR was 26.1% for the combination group vs. 17.5% for Lenvatinib. Severe AEs occurred in 62.5% of the combination arm and 57.5% of the single agent arm. Therefore, the study did not meet the pre-specified statistical significance for the primary endpoints [64].

#### 2.2.4. Nivolumab and Lenvatinib

A phase Ib clinical trial, Study 117, tested the combination of Nivolumab (240 mg NIV IV Q2W) plus Lenvatinib (bodyweight ≥ 60 kg: 12 mg/day; <60 kg: 8 mg/day) PO QD for 30 treatment-naive aHCC patients. Tolerability and safety were the primary endpoints while ORR was a secondary endpoint. All patients experienced AEs including palmar-plantar erythrodysesthesia (56.7%) and dysphonia (53.3%); however, they were manageable. Treatment discontinuation due to Lenvatinib plus Nivolumab occurred in 6.7% and 13.3% of patients, respectively. ORR was 76.7%. This combination proved well-tolerated with promising anti-cancer effects [65].

#### 2.2.5. Avelumab and Axitinib

VEGF Liver 100 is a phase Ib trial evaluating Avelumab (Bavencio) (10 mg/kg intravenously every 2 weeks) in combination with Axitinib (Inlyta) (5 mg orally twice daily) for 22 naïve aHCC patients. Avelumab is a human anti–PD-L1 IgG1 mAb while Axitinib is a TKI that selectively inhibits VEGFR 1–3. Hypertension (50.0%) and hand–foot syndrome (22.7%) were the most common grade 3/4 AEs while hypothyroidism (31.8%) and hyperthyroidism (13.6%) were the most frequent immune-related AEs. No treatment-related discontinuation was observed. ORR was 13.6%. At cutoff data, OS data were immature. Follow-up is ongoing [66].

#### 2.2.6. Camrelizumab and Apatinib

An open-label, multicenter, phase II study (RESCUE) was designed to define the safety and efficacy of Camrelizumab (AiRuiKa) (200 mg for bodyweight ≥ 50 kg or 3 mg/kg for bodyweight < 50 kg every 2 weeks), an anti-PD-1 antibody, in combination with Apatinib (Rivoceranib) (250 mg daily), an anti-VEGFR2, as first- or second-line therapy for patients affected by HBV-related aHCC. The survival benefit was observed both in first-line and second-line groups. The 12-month survival rate, PFS, and ORR were 74.7% vs. 68.2%, 5.7 vs. 5.5 months, and 34.3% vs. 22.5% in first- vs. second-line groups, respectively. Grade ≥ 3 AEs were experienced by 77.4% of patients: hypertension (34.2%) was the most frequent [67].

Based on these data, a phase III trial (NCT03764293) is ongoing to compare this combination with Sorafenib.

### 2.3. PD-1/PD-L1 Inhibitors Plus Other Immunotherapies

Tumor cells can avoid the immune system using several ways; therefore, the combination of ICIs with different mechanisms of action may represent an interesting treatment strategy [69]. Moreover, the inhibition of the B7-CTLA-4 pathway from anti-CTLA-4 antibody favors the anticancer effects through the increase of the activated CD8+ T cell level in lymph nodes and consequently into TME [70]. On the other hand, only when the required CD8+ T cells are present in TME, the inhibition of the PD-1/PD-L1 pathway activates tumor immunity [70]. Furthermore, the anti-CTLA-4 antibody can attenuate Treg cells in the immunosuppressive TME [69].

Table 3 summarizes all the combinations of PD-1/PD-L1 inhibitors with other ICIs that have been tested for the treatment of advanced HCC.

#### 2.3.1. Nivolumab and Ipilimumab

CheckMate 040 was the first clinical trial that evaluated the safety and efficacy of an ICIs combination for the treatment of aHCC [71]. It is a phase I/II study that tested Nivolumab plus Ipilimumab for 148 patients previously treated with Sorafenib. The enrolled population was randomized into three dosing arms (A: Nivolumab 1 mg/kg plus Ipilimumab 3 mg/kg every three weeks for 4 cycles; B: Nivolumab 3 mg/kg plus Ipilimumab 1 mg/kg every three weeks for 4 cycles. Subsequently, the A and B groups received Nivolumab 240 mg intravenously every two weeks. C: Nivolumab 3 mg/kg every two weeks plus Ipilimumab 1 mg/kg every six weeks). The best results were obtained in the arm that received the highest ipilimumab dose (group A) with a survival of 22.8 months and ORR of 32%. In this group, the highest complete response rate was also observed. However, group A also experienced the highest incidence of immune-related AEs (94%) although they were easily managed through the administration of corticoids. Grade 3/4 AEs were reported in 25% of patients.

On this basis, the combination regimen of Nivolumab plus Ipilimumab was approved by the FDA as second-line treatment in aHCC.

In addition, a meta-analysis documented the superiority of this second-line combination in terms of OS and PFS compared to Regorafenib (160 mg), Nivolumab (3 mg/kg), and Cabozantinib (60 mg) as single agents for patients affected by aHCC.

Finally, CheckMate 9DW (NCT04039607) is an ongoing phase III trial that was designed to test the clinical efficacy of Nivolumab plus Ipilimumab with respect to Sorafenib or Lenvatinib as first-line therapy for this set of patients.

#### 2.3.2. Tremelimumab and Durvalumab

Other studies confirmed the dose-dependence efficacy of anti-CTLA4 antibodies. For example, an open-label randomized phase I/II clinical trial analyzed the efficacy and safety of Tremelimumab (anti-CTLA-4) plus Durvalumab (anti-PD-L1) as a second-line treatment for aHCC patients. The results showed that the group treated with the highest dose of Tremelimumab (Tremelimumab 300 mg plus Durvalumab 1500 mg followed by durvalumab every 4 weeks) obtained the best OS (18.7 months) and ORR (24%) with good tolerability (Grade ≥ 3 AEs in 37.8% of patients).

HIMALAYA was an open-label, multicenter phase III study concerning untreated aHCC patients that tested Tremelimumab (a single dose of 300 mg) plus Durvalumab (1500 mg every 4 weeks), Durvalumab single agent (1500 mg every 4 weeks), and Sorafenib (400 mg twice daily) [72]. Enrollment in Tremelimumab 75 mg plus Durvalumab arm was stopped after a planned analysis showed no difference with respect to Durvalumab single agent. The primary endpoint was OS for the combination group compared to Sorafenib while the secondary endpoint was OS non-inferiority of Durvalumab to Sorafenib single agents. The study confirmed the superiority of the higher Tremelimumab dose regimen compared to Sorafenib in terms of OS (16.4 vs. 13.8 months, *p* = 0.0035) and the non-inferiority of Durvalumab single agent with respect to Sorafenib. Median PFS was 3.8 months, 3.7 months, and 4.1 months while ORR was 20.1%, 17%, and 5.1%, respectively. Therefore, these results significantly support the use as first-line therapy of Tremelimumab 300 mg single dose plus Durvalumab 1500 mg regimen followed by Durvalumab every 4 weeks for aHCC. In this group, grade 3/4 AEs were observed in 26% of patients unlike 12.9% of patients in the Durvalumab group and 36.9% of patients in the Sorafenib group. Therefore, this study put evidence that the combination of Tremelimumab (a single dose of 300 mg) plus Durvalumab (1500 mg every 4 weeks) is superior to Sorafenib with a favorable benefit-risk profile, suggesting a novel therapeutic first-line strategy for this patient population.

## 3. Adoptive Cell Transfer

While ICIs act by restoring or increasing the natural immune responses, adoptive cell transfer (ACT) treatment leads to new different immune responses. This therapy depends on the expansion and modification in vitro of allogeneic or autologous immune cells and their transfer back to patients [73]. On the other hand, ACT treatment is based on the identification in vitro of specific TAAs that can trigger an efficient immune response against cancer cells. This strategy allows the high specificity and individualization of ACT treatment [74].

T cell receptor (TCR)-engineered T cells, chimeric antigen receptor T cells (CAR-T cells), tumor-infiltrating lymphocytes (TILs), and cytokine-induced killer cells (CIKs) are examples of ACT therapies that are showing encouraging anticancer activities against HCC [73,75].

Modified TCR-engineered T cells can recognize and bind the TAAs of cancer cells and MHC of APCs. Interestingly, these modified T cells can also recognize antigens that are not confined to the membrane, unlike conventional T cells [76,77]. Some in vitro and in vivo studies demonstrated that these modified cells proved to be efficient for the treatment of HCC with a safe profile of toxicity. In detail, TCR-engineered T cells with HBV antigens as targets have shown antitumor activities in HBV-related HCC. On the other hand, TCR-engineered T cells specific to glypican-3 (GPC-3) or AFP proved to inhibit tumor progression [78,79]. Currently, several trials are ongoing to test these modified cells specific to various TAAs such as HBV antigen (NCT03899415) and AFP (NCT03971747, 04368182, 03132792).

CAR-T cells can recognize and destroy liver cancer cells by targeting specific TAAs without MHC restriction. This mechanism might represent an efficient strategy for preventing the cancer immune escape caused by MHC down-regulation [80]. Some prospective phase I clinical trials evaluated autologous GPC-3–CAR-T cell therapy in patients with GPC-3 positive aHCC [81]. The results put evidence of a survival benefit; in particular, the OS rates were 50.1%, 42.0%, and 10.5% at 6 months, 1 year, and 3 years, respectively. In terms of toxicity, this therapy was safe with only one patient suffering from grade 5 cytokine release syndrome [81]. Among patients with CD133-positive unresectable HCC, more than 50% of them experienced a PFS of 6.8 months and an OS of 12 months after CD133-CAR-T cells reinfusion [82].

As regards CIKs and TILs, clinical trials are also undergoing with interesting experimental findings [76,83]. Based on these data, ACT treatment might become a valid alternative strategy for the treatment of these patients.

## 4. Vaccines

In the last decades, several therapeutic vaccines have been experimented involving DCs, oncolytic viruses, and peptides.

DC vaccines are a type of cellular vaccine that can stimulate a strong anticancer immune response through the involvement of effector T cells [84]. The latter, in turn, act by killing cancer cells with the consequent release of TAAs further fueling the immune response. Phase I and II clinical studies showed that DCs pulsed with tumor lysis led to a modest antitumor efficacy (mean survival of 5.5 months) and a safe profile of toxicity in patients affected by aHCC [85,86].

Other clinical studies tested the safety and efficacy of peptide vaccines, using AFP, GPC-3185, and multidrug resistance-associated protein 3 [87,88,89]. However, the results of these trials documented only a modest antitumor effect probably due to the limited population and design of the studies.

Oncolytic virus vaccines consist of gene-modified viruses with a specific cellular tropism. These oncolytic viruses can replicate inside their target tumor cells and kill them. The subsequent release of TAAs further stimulates the antitumor immune response [90,91]. TRAVERSE was a randomized phase II study that tested JX-594, a modified poxvirus, inserted into the human granulocyte–macrophage colony-stimulating factor gene as a treatment for aHCC patients [92]. This study showed that the experimental therapy with high doses led to a longer OS than low doses (OS 14.1 vs. 6.7 months). Subsequently, the PHOCUS phase III trial was designed to compare JX-594 plus Sorafenib with Sorafenib alone [93]. The results demonstrated that the modified poxvirus did not improve the anticancer effect. Currently, other clinical trials are ongoing to compare JX-594 with Nivolumab (NCT03071094) or other ICIs [94].

## 5. Notch Signaling Pathway: The Role in HCC Development and Response to Cancer Treatments

The Notch signaling pathway is involved both in human embryonic cell development and the maintenance of adult stem cells through cell-to-cell communication through four Notch receptors (Notch1, Notch2, Notch3, and Notch4) [95,96,97]. Further evidence showed the role of this pathway in different human diseases including cancer [98,99]. In detail, dysregulation of Notch receptors has been found in various human cancers where they are involved in growth arrest, proliferation, and differentiation [100].

There is mounting evidence that Notch signaling pathway might play a pivotal role in HCC development. Interestingly, almost 80% of human HCC specimens have a high level of Notch expression than the adjacent normal tissues. In detail, HCC tissues express a higher level of Notch1 in the cytoplasm and Notch4 in the nucleus and a low level of Notch2 in the cytoplasm with respect to non-tumor-adjacent tissues while no difference was observed between Notch3 and Notch4. Most of the literature data regard the role of Notch1 and Noth3 in HCC development [101]. In vitro studies demonstrated that increased Notch1 might promote liver carcinogenesis through the Wnt/β-catenin pathway. Additionally, in vivo studies support that Notch1 signaling might promote HCC carcinogenesis in animal models [102,103]. Moreover, Notch1 mRNA levels are strictly correlated with HCC TNM staging. In detail, HCC patients with TNM Stage III–IV and tumor venous invasion had higher expression levels of Notch1 with respect to those patients with TNM Stage I–II disease and/or without tumor venous invasion. In this regard, other studies showed as the downregulation of Notch1 inhibited the invasion of HCC preventing HCC metastasis both in vitro and in vivo [104,105]. With regards to Notch2 and Notch4, some studies found that they might be involved in cancer aggressiveness and metastasis [106,107,108,109]. Other studies documented the important role of these receptors in the proliferation of hepatoblasts [110].

Regarding Notch3, it is a potential marker of stem/progenitor cells (FLSPCs) and is able to regulate their differentiation into hepatocytes [111]. This involvement in differentiation leads to assume that it might have a role in HCC development. To this regard, approximatively 78% of early HCCs presents Notch3 abnormal accumulation [107]. Other studies reported that Notch3 gene is the Notch pathway member with the highest expression in HCC tissues with respect to normal liver tissue [112]. However, in contrast, some studies showed that there is no difference in terms of Notch3 expression between HCC tissues and adjacent normal liver cells [113]. This suggests that TME factors are probably involved in HCC carcinogenesis. Furthermore, some studies documented the possible Notch pathway role in the genesis of chemoresistance. For example, a recent study regarding ovarian cancer showed that Notch3 increased resistance to platinum-based chemotherapy [114]. Similarly, a study about prostate cancer documented that knocking down Notch1 sensitized the cells to docetaxel [115]. With regards to HCC, Notch3 ablation enhances the apoptotic effect of doxorubicin in tumor models [116]. In addition, in vitro and in vivo studies reported that Notch3 inhibition exacerbates the efficacy of sorafenib in HCC [117]. Ma et al. evaluated the correlation between a notchScore based on 10 Notch pathway-related genes (NPRGs) and the clinical characteristics of HCC patients. The results showed that a high notchScore was an independent negative prognostic factor associated with severe OS. Moreover, a high score was associated with higher pathological stages, immune cells, immune score, and ICPs. Therefore, the authors propose that a high notchScore might have a prognostic value and correlate with sensitivity to immunotherapy in HCC [118].

Finally, more and more literature data confirm the important role of the Notch signaling pathway in cancer development, including HCC. Further data suggest that the dysregulation of Notch genes might be involved in resistance or sensitivity to conventional cancer therapy, including immunotherapy of therapy with TKIs.

## 6. Conclusions and Future Perspectives

Patients affected by aHCC have a poor prognosis; systemic therapies are the only treatment options to obtain a survival improvement. In the last decades, new molecular targeted therapies have been developed such as Sorafenib, Lenvatinib, Cabozantinib, Regorafenib, and Ramucirumab, but the prognosis has remained poor [36,119,120].

Given the common state of autoimmune tolerance in the hepatic microenvironment, the chronic inflammation due to cirrhosis and hepatitis B or C in which HCC often develops, and the synergistic effects deriving from combination therapies, various ICIs have been evaluated for the treatment of this tumor during the last years, both as single agents both in association with antiangiogenetic drugs or other ICIs (anti-PD-1/PD-L1 and anti-CTLA-4 antibodies). According to the reported data, single-agent treatments did not lead to favorable outcomes, probably due to the complexity of the TME in HCC. Conversely, combination therapies demonstrated an important survival benefit. Important progress in terms of survival has been obtained for these patients thanks to the efficacy demonstrated, for example, by the combination of Atezolizumab and Bevacizumab, the new standard of care. However, the continuous study of TME allows us to deepen our knowledge about the different mechanisms of immune escape that make HCC resistant to immunotherapies. Therefore, many other pathways may exist to be used as targets of novel drugs with the aim to improve anti-cancer activity. Consequently, more and more clinical trials will be necessary to evaluate the synergistic effects of different antitumor mechanisms. In terms of toxicity, ICIs have proven to be safe drugs; the most common and important AEs are fewer, immune-related diarrhea, gastrointestinal disorders, skin reactions, immune-related pneumonia, and liver and kidney toxicity. At the same time, immunotherapy management should be carefully monitored so that the detection and treatment of AEs are performed with the right timing, avoiding unnecessary treatment interruptions or deterioration of quality of life. In this regard, future clinical trials will have to evaluate the optimal dosing schedules and therapy duration with the aim to ameliorate AEs or toxicities deriving from novel rational combination regimens [121]. Moreover, a better knowledge of underlying mechanisms is required for improving the management of AEs as well as choosing the next treatment. Furthermore, a portion of aHCC patients do not experience a clinical benefit from ICI or develop primary or secondary resistance to them. There is little information about the predictors of response to immune-based therapies in these patients. Numerous studies have evaluated some tumor characteristics, such as intact IFN-y signaling, high tumor mutation burden, presence of ICPs, and high levels of TILs, which have demonstrated a positive clinical response to immunotherapy [29,54,63,64,68]. Almost 40% of HCCs have the constitutive activation of WNT/β-catenin signaling due to relevant gene mutations. These patients have negative data in terms of DCR and PFS from anti-PD1 therapies [122,123,124,125,126]. In this regard, it has been demonstrated that the WNT/β-catenin pathway favors the resistance to ICIs through the transcriptional repression of chemokine genes, leading to a consequent failure to prime and recruit CD8+ T cells. Another important pathway regarding immune regulation is the phosphatase and tensin homologue-signal transducer and activator of transcription 3 (PTEN-STAT3). In detail, PTEN knock-down decreases the killing of tumor cells by T cells through the indirect activation of STAT3. It is a transcription factor involved in tumor angiogenesis, metastasis, resistance to apoptosis, and immune escape through the regulation of the Toll-like receptors and interferon-inducible genes expression [126,127]. Approximately, 60% of HCCs have a STAT3 activation that is also associated with a poor prognosis.

Thus, the necessity of finding some predictive biomarkers to select those HCC patients might benefit more from a specific therapy. Currently, AFP is the only valuable biomarker to guide treatment and predict prognosis for HCC; however, many other potential biomarkers for HCC have been evaluated in different studies. For example, prothrombin induced by vitamin K absence-II (PIVKA-II) showed higher sensitivity and specificity than AFP, but it has not yet been widely employed in clinical practice probably due to the limitation of geographic populations and underlying liver disease [128]. Of note, multiple biomarkers in concert may also exhibit superiority over biomarkers used alone [129]. This is exemplified by the multi-marker panels consisting of AFP, PIVKA-II, and other biomarkers. Therefore, further large-scale and international multicenter prospective clinical studies are necessary to test the clinical role of the different biomarkers. Immunoscores have been designed for HCC patients to help determine patient recurrence, survival rates and response rates to immunotherapies [67]. In this regard, the CRAFITY (C-reactive protein (CRP) and alpha-fetoprotein (AFP) in immunotherapy) score for HCC was associated with survival and radiologic response among patients receiving PD-1 immunotherapy. Additionally, the etiology of liver disease may be a guide for the choice of aHCC treatment [32]. In detail, a recent meta-analysis reported that immunotherapies may be more effective among patients with viral etiology-related HCC compared to NALFD-related HCC [71]. In conclusion, drug combination regimens based on a better knowledge of the biological mechanisms of HCC in TME and personalized treatments according to specific biomarkers are the targets to reach in the near future with the aim to achieve a better prognosis and quality of life for aHCC patients.

## Figures and Tables

**Table 1:** PD-L1 inhibitors as single agents for the treatment of advanced HCC.

PD-1/PD-L1 Inhibitors	Trial	Comparison	Setting	OS	PFS/TTP *	ORR/DCR **	Grade 3–4 AEs
Nivolumab (Opdivo)	CheckMate-040 (Phase I–II) [40]	/	≥Second-line	/	3.4 months *	15–20% 58–64% **	25%
Nivolumab (Opdivo)	CheckMate-459 (Phase III) [41]	Sorafenib	First-line	16.4 vs. 14.7 months; HR = 0.85; *p* = 0.0752	/	15% vs. 7%	22% vs. 49%
Pembrolizumab (Keytruda)	KEYNOTE-224 (Phase II) [42]	/	Second-line	12.9 months	4.9 months	17%	25%
Pembrolizumab (Keytruda)	KEYNOTE-240 (Phase III) [43]	Placebo	Second/Third-line	13.9 vs. 10.6 months; HR, 0.781; *p* = 0.0238	3.0 vs. 2.8 months HR = 0.718; *p* = 0.0022	18.3% vs. 4.4% *p* = 0.00007	52.7% vs. 46.3%
Pembrolizumab (Keytruda)	KEYNOTE-394 (Phase III) [44]	Placebo	Second-line	13.6 vs. 13.0 months; HR 0.79, *p* = 0.0180	2.6 vs. 2.3 months; HR 0.74, *p* = 0.0032	13.7% vs. 1.3%	14.4% vs. 5.9%
Camrelizumab (SHR-1210)	Phase II [45]	/	Second-line	74.4% alive at six months	/	14.7%	22%
Tislelizumab (BGB-A317)	RATIONALE-301 (Phase III) [46]	Sorafenib	First-line	15.9 vs. 14.1 months; HR: 0.85	/	25% vs. 10.3%	48.2% vs. 65.4%
Durvalumab (Imfizi)	Phase I–II [47]	/	Second-line	19.3 vs. 13.2 months	/	25% vs. 10.3%	24%

Abbreviations: Overall Survival (OS); Progression Free-Survival (PFS), Time to Progression (TTP) *, Objective Response Rate (ORR); Disease Control Rate (DCR) **.

**Table 2:** PD-L1 inhibitors with angiogenetic drugs for the treatment of advanced HCC.

Combination	Trial	Comparison	Setting	OS	PFS/TTRP *	ORR/DCR **	Grade 3–4 AEs
Bevacizumab (Avastin) + Atezolizumab (Tecentriq)	IMBrave150 (Phase III) [62]	Sorafenib	First-line	19.2 vs. 13.4 months; HR = 0.66; *p* = 0.0009	6.8 vs. 4.3 months; HR 0.59	43% vs. 32%; *p* = 0.002 **	56.5% vs. 55.1%
Sintilimab (Tyvyt) + Bevacizumab (IBI305)	ORIENT-32 (Phase II–III) [63]	Sorafenib	First-line	Median not reached vs. 10.4 months; HR 0.57; *p* < 0.0001	4.6 vs. 2.8 months, HR 0.56, *p* < 0.0001	/	14% vs. 6%
Pembrolizumab (Keyruda) + Lenvatinib (Lenviva)	LEAP002 (Phase III) [64]	Sorafenib	First-line	21.2 vs. 19 months HR 0.840, *p* = 0.0227	/	26.1% vs. 17.5%	62.5% vs. 57.5%
Nivolumab (Opdivo) + Lenvatinib (Lenviva)	Study 117 (Phase Ib) [65]	/	First-line	/	/	76.7%	55%
Avelumab (Bavencio) + Axitinib (Inlyta)	VEGF Liver 100 (Phase Ib) [66]	/	First-line	/	/	13.6%	50%
Camrelizumab (AiRuiKa) + Apatinib (Rivoceranib)	RESCUE (Phase II) [67]	/	First/second-line	The 12-month survival rate, was 74.7% vs. 68.2% in first- vs. second-line groups, respectively.	5.7 vs. 5.5 months in first- vs. second-line groups, respectively	34.3% vs. 22.5% in first- vs. second-line groups, respectively	77.4%

Abbreviations: Overall Survival (OS); Progression Free-Survival (PFS), Time to Radiological Progression (TTRP) *, Objective Response Rate (ORR); Disease Control Rate (DCR) **.

**Table 3:** PD-L1 inhibitors with other immunotherapies for the treatment of advanced HCC.

Combinations	Trial	Comparison	Setting	OS	PFS/TTRP *	ORR/DCR **	Grade 3–4 AEs
Nivolumab (Opdivo) + Ipilimumab (Yervoy)	CheckMate 040 (Phase I–II) [71]	Three different dosing arms	Second-line	22.8 months	/	32%	25%
Nivolumab (Opdivo) + Ipilimumab (Yervoy)	CheckMate 9DW (Phase III)	Sorafenib or Lenvatinib	First-line	Ongoing	Ongoing	Ongoing	Ongoing
Tremelimumab (a single dose of 300 mg) + Durvalumab (1500 mg every 4 weeks)	HIMALAYA (Phase III) [72]	Sorafenib	First-line	16.4 vs. 13.8 months *p* = 0.0035	3.8 vs. 4.1 months	20% vs. 5.1%	26% vs. 37%

Abbreviations: Overall Survival (OS); Progression Free-Survival (PFS), Time to Radiological Progression (TTRP) *, Objective Response Rate (ORR); Disease Control Rate (DCR) **.

## Abbreviations

Adoptive Cell Transfer	ACT
Adverse Event	AE
Angiopoietin 2	ANG2
Antigen-Presenting Cell	APC
Barcelona Clinic HCC	BCLC
Chimeric Antigen Receptor T Cell	CAR-T Cell
Cytokine-Induced Killer Cell	CIK
Cytotoxic T-Lymphocyte	CTL
Cytotoxic T Lymphocyte-Associated Antigen 4	CTLA-4
Dendritic Cell	DC
Disease Control Rate	DCR
ECOG Performance Status	ECOG
Hepatocellular Carcinoma	HCC
Immune Checkpoints	ICPs
Intercellular adhesion molecule 1	ICAM1
Lymphocyte Activation Gene-3	LAG-3
Major Histocompatibility Complex Class 1	MHC-1
Median Time to Progression	mTTP
Monoclonal Antibody	mAb
Natural Killer Cell	NK
Notch pathway-related genes	NPRGs
Objective Response Rate	ORR
Overall Survival	OS
Programmed Cell Death 1	PD-1
Progression-Free Survival	PFS
Regulatory T Cell	Treg
Stem/progenitor cells	FLSPCs
T Cell Receptor	TCR
T-Cell Immunoglobulin Mucin-3	TIM-3
Transforming Growth Factor-Β	TGF-Β
Tumor-Associated Antigen	TAA
Tumor-Infiltrating Lymphocyte	TIL
Tumor-Associated Macrophage	TAM
Tumor Microenvironment	TME
